# Application of transcritical CO_2_ heat pumps to boiler replacement in low impact refurbishment projects

**DOI:** 10.1016/j.heliyon.2024.e26929

**Published:** 2024-03-06

**Authors:** William Lambert, Zahir Dehouche

**Affiliations:** Department of Mechanical and Aerospace Engineering, College of Engineering, Design and Physical Sciences, Brunel University London, UB8 3PH, United Kingdom

**Keywords:** CO_2_, Transcritical, Heat pump, Space heating, Simscape, Defrosting, Radiator pulsing

## Abstract

80% of current UK housing stock is expected to still be in use in 2050. Difficult, intrusive and expensive, refurbishment measures are required to achieve the level of insulation required for current low temperature heat pumps. Transcritical CO_2_ heat pumps can achieve higher efficiencies, with higher output temperatures, than current, Carnot limited, synthetic gas heat pumps, with less environmental impact. Widely deployed in water heating and supermarket chilling systems, CO_2_ heat pumps need heating return temperatures of 30 °C or less to function effectively. This has impeded their adoption with hydronic heating systems which have high return temperatures.

This study identified system modifications external to the refrigeration cycle that address return temperatures. It modelled a transcritical CO_2_ air source heat pump with a hydronic heating system in a solid wall semi-detached house. Full year system coefficients of performance over 3 were achieved in four UK locations by using space heating return fluids to defrost the air source heat exchanger and to pre-heat inlet water, recovering any remaining excess return fluid heat as a source for the heat pump. Solar panels boosted this to 5.1. The levelized cost of energy for the system was calculated (with heat pump grant) at 22p/kWh, lower than a gas boiler, with 9.45 tonnes CO_2_ emission savings over a fifteen-year life.

## Nomenclature

ASHE: Air Source Heat Exchanger
EPC: Energy Performance Certificate
ADMD: After Diversity Maximum Demand
DHW: Domestic Hot Water
COP: Coefficient of Performance
LCOE: Levelized Cost of Energy (p/kWh)
PCM: Phase Change Material
UK: United Kingdom

## 1 Introduction

The Intergovernmental Panel on Climate Change warns that without “immediate and deep … …emission reductions across all sectors” limiting global warning to 1.5 °C is beyond reach [1]. The UK, alongside many other nations, has set a target to be carbon neutral by 2050. This requires the near elimination of global warming greenhouse gas emissions.

While much progress is being made in electricity generation and transport sectors, many other sectors are proving hard to de-carbonise. In its 2019 report “UK housing: Fit for the future?” (p27) [2] the UK Committee for Climate Change (UKCCC) estimated that 15% of the UK's current greenhouse gas emissions come from space and water heating in homes. The UK housing stock ranked a low 11, out of 15 European countries, on energy efficiency and 80% of this stock will still be in operation by 2050 [3].

Whilst district heat networks may be able to address requirements in high density city areas (especially where waste heat from industrial processes is available), low temperature heat pumps often require intrusive insulation and plumbing upgrades, to provide adequate heat on cold days [4]. There are few effective solutions currently available for old, low density, housing. Building owners are left with expensive and inconvenient options and so decarbonization of heating is proceeding slowly.

CO_2_ was used in some of the first heat pumps developed in the nineteenth century. It faded into obscurity in the twentieth century with the arrival of CFCs and other synthetic refrigerants. Its use was re-examined by Lorentzen in 1994, who published a seminal paper on the revival of CO_2_ as a refrigerant, highlighting the potential the transcritical cycle [5]. CO_2_ has a far lower global warming and ozone depletion impact than commonly used synthetic refrigerants and, used in its transcritical cycle, can maintain heat pump performance at lower source temperatures and higher delivery temperatures than most synthetic refrigerants.

Since 1994 the technology has advanced. Over 5 million ‘Eco-Cute’ transcritical CO_2_ heat pumps have been sold in Japan for domestic water heating [6]. 14% of food industry refrigeration in Europe is now CO_2_ driven [7]. High efficiency, compact, applications in electric vehicles are showing promise [8]. Recently a water and space heating system has been installed in Wolfson College, Oxford [9].

**Fig. 1:**
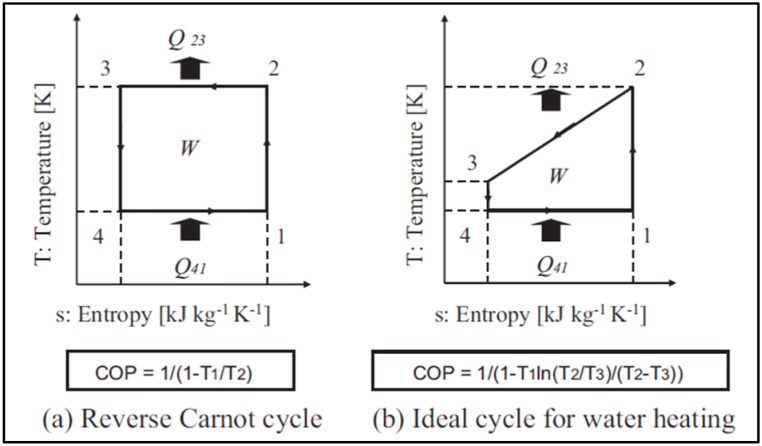
Carnot vs Transcritical Ideal COPs.

Transcritical CO_2_ heat pumps are able to achieve coefficients of performance (COP) over 4 while delivering temperatures over 60 °C [10]. Saikawa and Koyama [11] show that the Carnot cycle limits do not apply (see Fig. 1). The transcritical cycle with a 20 °C source, heating water from 20 °C to 80 °C, has an ideal COP of 11.1 vs only 5.9 for the Carnot cycle.

While the heat pumps operate at high pressure, increasing some costs, the high density of compressed CO_2_ offers the opportunity for smaller components and so low material costs if mass produced [5].

Many older homes in the UK are fitted with radiators designed for 82 °C outflow and 71 °C return temperatures [12]. Where condensing boilers have been fitted, return temperatures are closer to 50 °C. These return temperatures are too high for the efficient operation of transcritical CO_2_ heat pumps.

### 1.1 UK retrofit and building demand

Lingard [3] studied the retrofit implications of heat pump deployment. He identified the limitations of the UK grid which has been built on the “after diversity maximum demand” (ADMD) assumption of 1.5 kW for domestic dwellings and examined how often a heat pump would exceed 1 kW (2/3 of ADMD). Using IES software, he modelled a pre-retrofit 3 bedroom solid wall semi-detached house which had an annual heating demand of 94.4 kWh/m^2^/yr and corresponded to an EPC rating of “D”.

He explored the contributions of different elements of UK Building Retrofit Regulations L1B (combining to achieve 43 kWh/m^2^/yr) and an enhanced retrofit with photovoltaic solar panels achieving 19 kWh/m^2^/yr. Solid wall insulation was the most significant intervention in the retrofits. Only in the enhanced retrofit did the heat pump exceed 1 kW for less than 5% of time.

There is no mention of the impact of COP variation. Lower COPs in winter could increase the high power periods for the heat pump and/or the retrofit insulation requirements.

To provide domestic hot water (DHW) further power is required, especially in cold periods when more DHW is used and when the water inlet starts at lower temperature. For these reasons, the challenge to make heat pumps grid friendly was probably underestimated by Lingard. His paper provides space heating demand data and an approach to retrofit that helps clarify requirements and opportunities.

The DHW requirements were measured by the Energy Savings Trust in 2008 [13] identifying hourly and annual demand patterns and incoming water supply temperatures. DHW demand is more constant than space heating requirements, peaking at about 5.6 kWh/day.

### 1.2 Transcritical CO_2_ heat pumps

Lorentzen's 1994 paper [5] sketched the emergence of ammonia, SO_2_ and CO_2_ from a plethora of naturally occurring early refrigerants. However, by the 1930s and 40s, he notes these were replaced by manufactured freons, promoted for their ‘complete safety and harmlessness to the environment’. Claims that Lorentzen notes had proved horribly wrong by the time he wrote his paper.

Dilshad et al. [7] review the rise and demise of F-gases as their benefits, and then climate damage, became clear, and how CO_2_ is emerging across continents as a preferred refrigerant. A common, naturally occurring substance, CO_2_ safety issues such as high pressure operation, high coefficient of expansion, asphyxiation risk, freeze burns, are well studied and Dilshad et al. summarise the mitigation approaches identified.

**Fig. 2:**
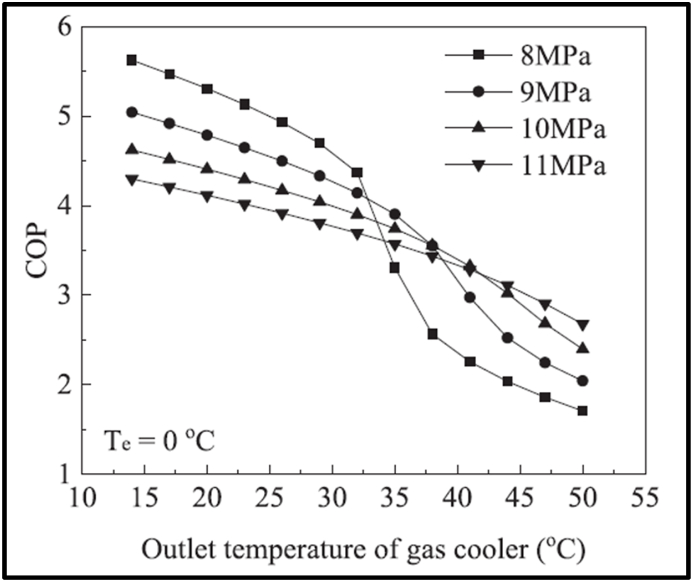
Reduction in COP as gas cooler CO_2_ output temperatures increase.

Significant research has been completed concerning the development and optimisation of transcritical CO_2_ heat pumps with several helpful literature reviews published [[14], [15], [16], [17]]]. However, the focus is often on refrigeration or water heating and only recently has there been significant research into using the heat pumps for space heating. Dai et al. [18] explore the benefits of dual compressors and ejectors in industrial heat recovery CO_2_ heat pumps. The potential benefits of transcritical heat cycles over Carnot cycles, illustrated in Fig. 1, and being achieved in current commercial CO_2_ heat pumps [10], are contingent on low CO_2_ gas cooler output temperatures (point 3 in Fig. 1). Yao et al. [19] show this in their graph of COP to gas cooler outlet temperature (cooled by the sink input fluid) reproduced in Fig. 2.

**Fig. 3:**
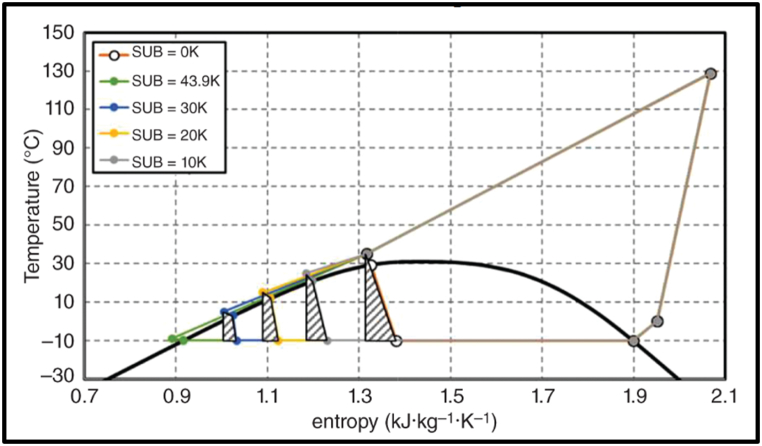
Zhang and Zamaguchi's Temperature – entropy diagram showing exergy losses.

Zhang and Zamaguchi [20] further explain that the hot compressed CO_2_ needs to be cooled to around 30 °C or less for expansion to work efficiently. The exergy losses for different cooling temperatures are shown in the shaded areas of their temperature – entropy diagram in Fig. 3.

Yang et al. [21] provide further insight in the context of water heating, showing how heating capacity decreases as the temperature of the water entering the gas cooler increases.

### 1.3 Controlling the gas cooler outlet

A number of different ideas have been identified in the literature that may help in achieving low gas cooler outlet temperatures while utilising the higher efficiencies and more manageable safety risks promised by the CO_2_ transcritical cycle. These all concern making full use of space heating return fluids and cooling the heat pump gas cooler input fluids.

• Wang et al. [22] propose combining high temperature radiators with low temperature underfloor heating;
• Embaye [23] showed that off/on pulsing radiators can increase energy efficiency by up to 25% without sacrificing comfort.
• Wang et al. [24] used phase change material (PCM) both to store high temperature heat and, separately, to regulate the heating return fluid and cool the gas cooler outlet.
• Dai, with different co-authors in 2019 and 2024 [25,26], employed dedicated mechanical subcooling using a second refrigeration cycle to cool the gas cooler outlet.
• Brodal and Jackson [27] explored cooling the PCM with the cold DHW inlet, pre-heating this for domestic hot water supply. They observed the heat pump COP falling from 4.5 to 4 as the heating ratio of space to water heating energy increased from 0.5 to 2.

Brodal and Jackson expected CO_2_ heat pumps to outperform R410A refrigerant systems at low heating to hot water ratios, with the cross over point between 0.6 and 1 depending on the specific implementations, but did not explore ways to increase effectiveness at the ratios above 2 common in residential buildings.

Frosting can reduce the COP of an air source heat pump by 40% or more at temperatures around 7 °C and relative humidity of 60% [28]. Defrosting of air source heat pump heat exchangers requires energy. Cheng et al. [29] reviewed defrosting methods concluding that defrosting is most frequently performed by reversing the heat pump, extracting heat from the heated area to defrost the heat exchanger, resulting in a cooling of the indoors and a gap in heat production. While Cheng et al. identified a number of alternative approaches, no studies were found that attempted to use the energy in space heating return fluids for defrosting (with the simultaneous benefit of cooling the fluid prior to its re-entry into the gas cooler).

Should the above techniques prove insufficient to bring the gas cooler outlet temperature down to below 30 °C, an efficient way to recycle low temperature heat into the heat pump is also required. While multi-cycle systems have been used to reheat heating return fluids (Zhang and Zamaguchi [20] page 283), no studies were found using single compressor systems. Using return heat as a source for the heat pump, simultaneously cooling it before passing it into the gas cooler, explored in this study, was not found in the literature reviewed.

### 1.4 Operating temperatures

Heat pumps operate most efficiently when the temperature difference between sources and sinks are minimised [11]. This involves operating the heat pump when ambient temperatures are higher (usually in the day rather than night) and so storage is required to supply heat to the building when it is needed (especially in the early morning when outside temperatures are often at their lowest).

Hydronic heating systems have traditionally been designed to operate with radiator temperatures above 50 °C. The more heat available above this temperature, the less change is required to radiators and plumbing, and the more heat can be used directly without less efficient recycling [4].

### 1.5 Solar boosting

One further approach to increase the COP of heat pump systems is solar boosting. Chen et al. [30] used solar thermal panels in combination with a CO_2_ heat pump, switching between the two systems to provide space and water heating. System COPs of 13.5 for the solar mode and 2.11 for the heat pump were recorded, but no seasonal combined COP figure was reported.

### 1.6 Environmental - economic analysis

While pricing data is commercially sensitive, quickly outdated, country dependent and strongly linked to manufacturing and order volumes, some academic and government research have been published. Recent papers by Dai et al. [18], Lingard [3] and NESTA [31] provide some estimates of typical heat pump component costs, although Dai et al. are focused on Chinese industrial heat pumps with Lingard and NESTA on Carnot cycle UK domestic scale systems.

This study builds a model to explore system design approaches to maintaining gas cooler outlet temperatures below 30 °C and achieving seasonal average system-wide COPs in the UK climate in excess of 3 with levelized costs competitive with gas boilers.

## 2 Methodology

This study takes a systems approach to the application of a transcritical CO2 heat pump to a semi-detached house based on Lingard [3]. It explores the impact of a number of innovative system modifications, external to the heat pump cycle, to recover low temperature heat and to achieve the gas cooler temperatures required for heat pump operation. The objective is a full year average COP in excess of 3, to be within reach of cost equivalence with a gas boiler.

The study is based on a simulation model built in the MATLAB Simscape environment. This was selected based on Ko et al. [32], who recommended ‘acausal’ software for modelling the transient effects of transcritical heat pump cycles. Available freely to academic users, Simscape was selected over Trnsys or EES.

Simscape solves multiple, simultaneous, differential equations within and between components to model the behaviour of physical circuits and systems. Standard components for different circuits (liquid, gaseous, thermal, mechanical) are interconnected in a graphical environment. While Simscape has a limited library of components, it provides a custom component environment for the development of components that can be used in its graphical modelling environment. The source code is available for many of the standard components, enabling rapid development of variants.

**Fig. 4:**
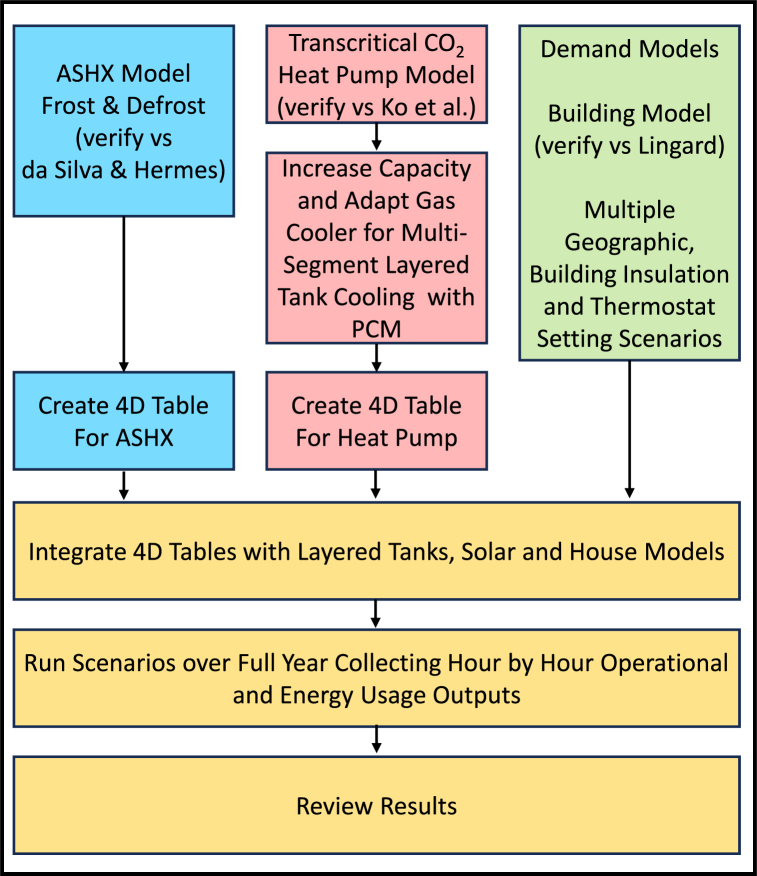
Model development flow chart.

The model was developed from established sub-system models, adapted and then integrated, to create a full system model. This was run over full year periods for multiple UK locations, building insulation levels and thermostat settings. The development flow chart is shown in Fig. 4.

Sub-systems were first developed, tested and calibrated against published data, before being integrated into the complete system. The control systems, less documented than the underlying physical processes, had to be developed from first principles. The sub-systems included are.

• CO_2_ transcritical heat pump;
• Air source heat exchanger with frosting and defrosting;
• Heat storage tanks with phase change materials;
• Building with hydronic heat distribution;
• Weather and demand model.

**Fig. 5:**
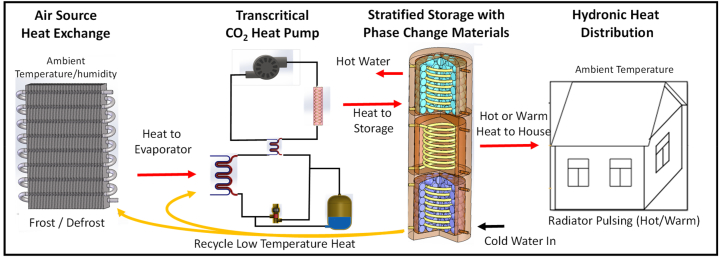
Overall model structure.

These were connected, as shown in Fig. 5, to explore the low exergy heat recycling.

### 2.1 CO2 transcritical heat pump

**Fig. 6:**
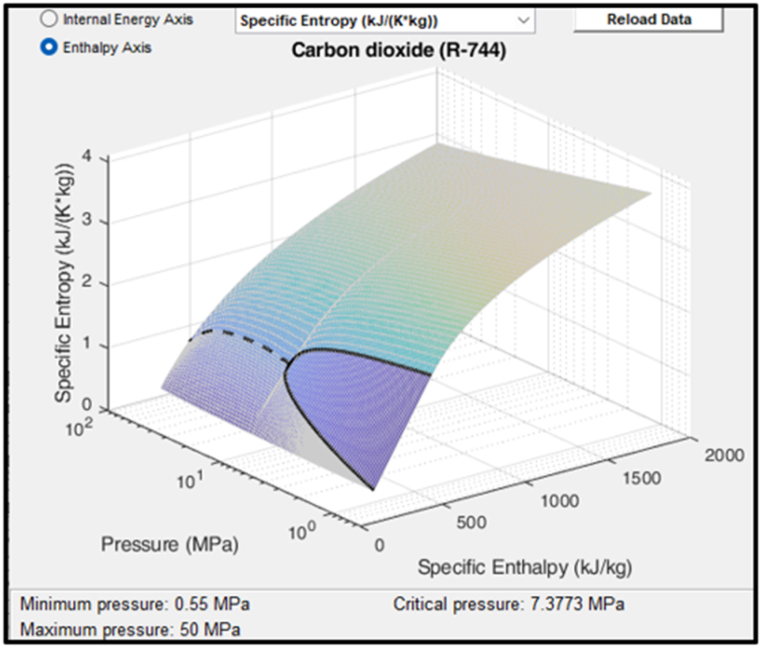
Simscape's CO_2_ properties.

The transcritical CO_2_ heat pump is based on the model developed in Simscape by Ko et al. [32]. It uses Simscape's standard CO_2_ properties block, a plot of its specific entropy values is shown in Fig. 6.

The model was first replicated as closely as possible and verified against Ko et al.‘s experimental data. It's COP output was between 1.6% and 7% out from Ko et al.‘s experimental data (a similar variance to that reported by Ko et al. for their model).

Ko et al.‘s model used evaporator temperatures of 15–20 °C and had heating capacities between 2.2 and 3.0 kW. This was insufficient to meet the anticipated heating demands of a medium sized home. The model relied on thermal masses for its heat source and sink, these behave differently to liquid circulation heat exchangers. A number of modifications were necessary.

• Increased heating capacity;
• Remodelling gas cooler to model temperature glide and increase capacity;
• Increasing capacity and temperature range of the evaporator and using water/glycol mix;
• Control systems to deliver set output temperatures under a full range of UK weather conditions.

To model temperature glide, the gas cooler was split into 5 stages, with each stage having corresponding heat transfer liquid pipes. To maintain simplicity and computational efficiency, increased capacity was achieved by modelling multiple CO_2_ pipes within a single water ‘shell’ pipe. A one-dimensional assumption with the Genic et al. [33] helical tube in shell heat transfer correlation was adopted. Chen Y [34] noted gas cooler performance discrepancies caused by using constant heat transfer properties for CO_2_ close to its critical point. Shorter segments were used where heat transfer was high and around the critical point.

Charge levels were found by Cho et al. [35] to have higher impact on COP with CO_2_ than with other refrigerants. Cho et al. researched cooling heat pumps but no studies for heating systems were found. A receiver was added to allow charge levels to be adjusted. Refrigerant charge is added, when required, into the low-pressure side of the system (ahead of the evaporator). CO_2_ can be extracted from the high-pressure side of the system. The receiver location follows Kim et al. [14] on the use of receivers.

Hundy et al. [36] provide insight into the multivariate complexity of heat pump control systems, optimisation of which is highly situation dependent. They provide some structure to assist development of control strategies. Controls were developed to seek robust transitions within 30 min through:

• Controlling suction vapour superheat;
• Achieving and maintaining a set gas discharge temperature and a set temperature for the heated water;
• Seeking a set discharge pressure;
• Adding or removing refrigerant charge to find the optimum operating level;
• Control the system through state changes and varying ambient conditions.

While most research has focussed on steady state optimisation, Xing et al. [37] provide some insight into the fluctuations and surges that hamper robust, predictable, operation across state changes. Combining these instabilities with the limitations of the discrete numerical processes used in Simscape's equation solving, requires all state transitions to be slowed and smoothed to maintain model operation.

While optimum gas cooler pressure, exit temperatures and suction superheat levels are reported in optimisation studies by Bruno et al. [16] and others, these are interim variables in a physical model, controlled indirectly. They are affected by the heat pump hardware, gas charge levels, expansion valve settings and compressor speeds as well as ambient and target output temperatures. Optimisation requires exploration of these primary settings alongside trade-offs on COP, capacity and power. Once a complete set of optimised settings have been determined, computational performance can be significantly improved by replacing the heat pump model with a performance map for optimal settings for the range of source and sink temperatures.

**Fig. 7:**
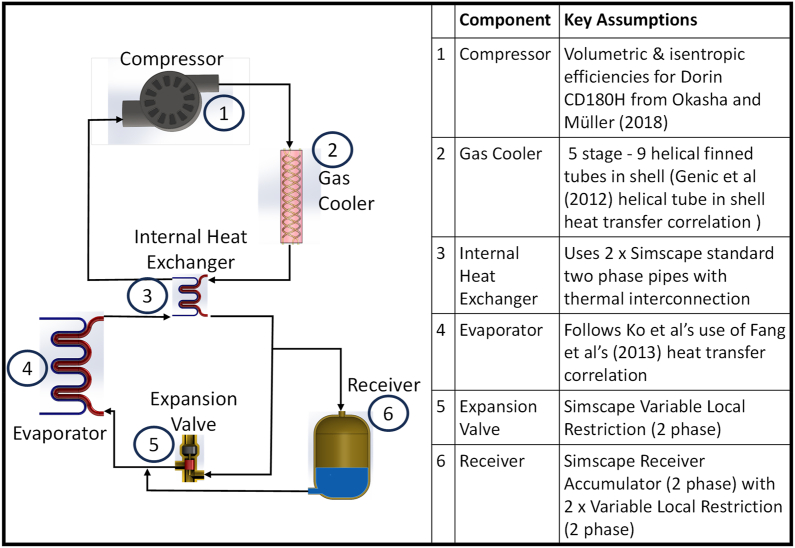
Heat pump model.

Fig. 7 shows the heat pump model with principal model assumptions.

### 2.2 Air source heat exchanger – frosting and defrosting subsystem

This subsystem integrates frosting and defrosting into a finned tube air source heat exchanger. Frost formation and defrosting was modelled using the approach and equations developed by da Silva and Hermes [38] following their initial papers with Melo [ [39,40]].

The heat source side uses a thermal liquid. This creates an extra heat exchange stage in the heat pump but allows multiple heat sources to be used. Also, it lowers the pressure and costs of the plumbing and creates efficient heat transfer for defrosting. The heating side models the transfer of heat from moist air through a frost layer, if present, into the heat exchanger. The frost models build and melt the frost layer in line with da Silva et al.‘s mathematical models. They incorporate the impact of the frost layer on heat transfer and on air flow.

The model had to adapt to the full range of UK air source heat pump operation. At higher temperatures no frost forms, but vapour does condense. At low humidity, neither frost formation nor condensation occurs. Zhu et al.‘s [41] suggestion to use a 6 °C transition temperature has been followed. It has been assumed that condensate falls off the heat exchanger immediately without affecting subsequent heat transfer. Although significant research exists on surface coatings and other frost reduction techniques these have not been incorporated into this model.

**Fig. 8:**
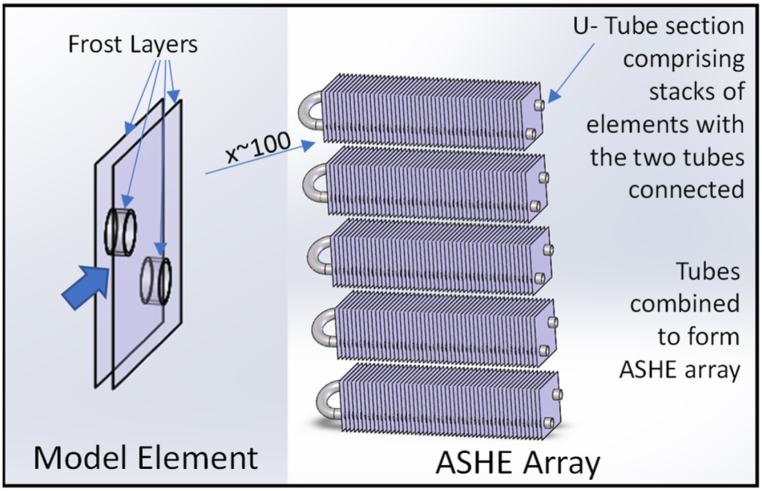
Air source heat exchanger model.

The model is based on the frost formation and dissipation in a single section of a two layer heat exchanger between two fins. This is multiplied by the number of fins to complete a Simscape component representing a single constant temperature tube spanning the air source heat exchanger (ASHE). As illustrated in Fig. 8, these components were replicated horizontally and vertically for the full ASHE.

The fin efficiency of a plane fin-tube heat exchanger with small diameter tubes was found to stay close to 0.9 when investigated by Lai et al. [42] so this value was adopted.

**Fig. 9:**
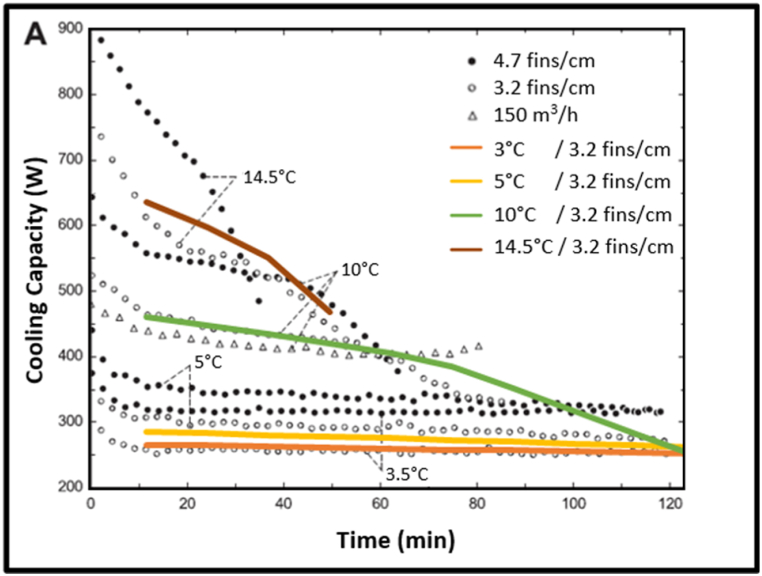
Cooling capacity of model (solid lines) with da Silva et al. (2011) results (empty circles).

The initial heat exchanger model was calibrated against da Silva et al.‘s [39] experimental results. The results are compared in Fig. 9. After calibration, the model was expanded. Combining multiple array elements with the heat pump model, led to convergence errors. A single array element was therefore used with the output multiplied to increase capacity.

Defrosting was modelled using the defrosting efficiency equations of da Silva and Hermes [37]. A heat transfer coefficient was estimated using a weighted average heat flux from Mohs and Kulacki [43] three defrosting periods.

### 2.3 Heat storage

**Fig. 10:**
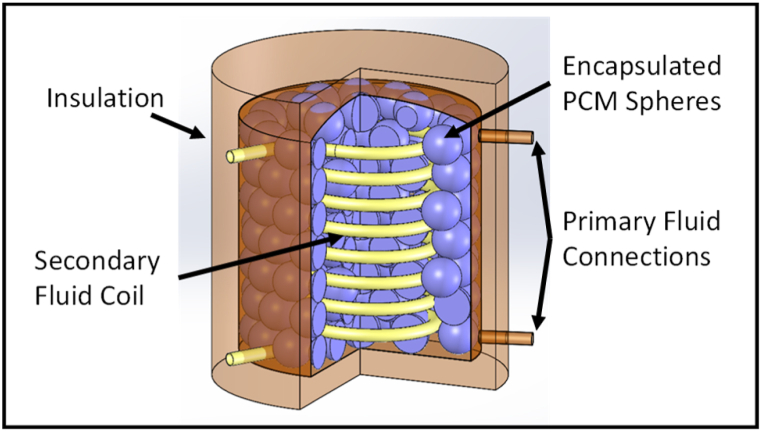
Heat storage tanks with PCM balls.

Fig. 10 shows the stratified tank, three of which are used in the system. The top tank is filled with phase change material (PCM) spheres encapsulated in a 2 mm layer of high density polyethylene (HDPE). The PCM spheres melt at around 65 °C. Heating fluid, 30% glycol/70% water by weight, fills the tank. This is heated in the gas cooler and provides the hot fluid for space heating. Domestic hot water (DHW) is heated in the helical coil (separation ensures no drinking water contamination).

The bottom tank is filled with lower temperature (∼18 °C melting) PCM spheres and heating fluid. DHW is preheated through the coil. The PCM helps to stabilise the fluid input to the heat pump gas cooler to around 20 °C. It also provides a heat source for the heat pump evaporator when outside temperatures drop too low for efficient operation or when there is an excess of medium temperature fluid. The packing densities of PCM spheres were determined through CAD models.

The middle tank, not containing PCM, allows greater temperature flexibility for the transition between top and bottom tanks.

**Fig. 11:**
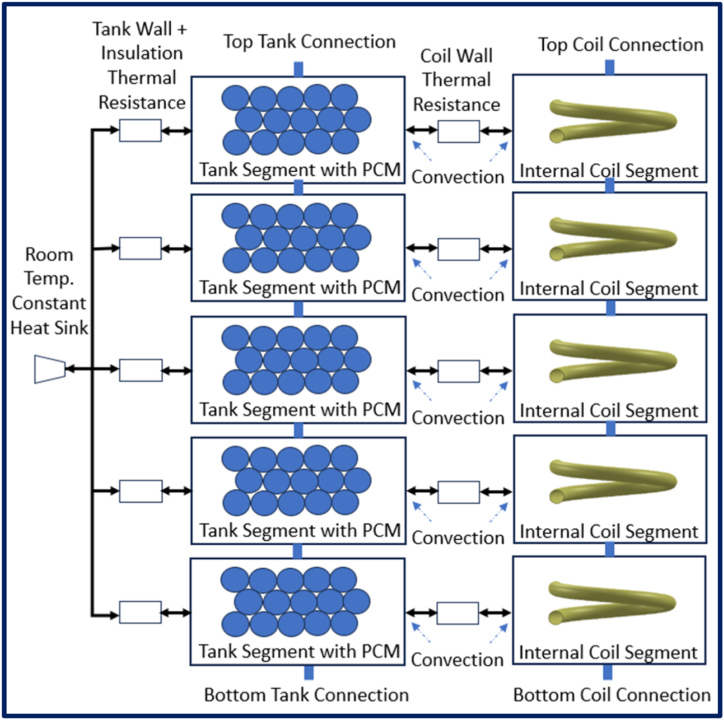
Cylindrical tank with PCM spheres and helical coil subsystem model.

The Simpscape Pipe model was adapted separately for the tank and the coil. Each tank was modelled in 5 layers to allow for stratification. The model components for a single tank are shown in Fig. 11. Heat transfer correlations were taken from Fernandez-Seara et al. [44] and Genic et al. [33].

The tank segment model covered.

• Fluid flows into the top and out of the bottom (carrying heat and momentum);
• Convective heat flows through the tank exterior to the interior of the insulation;
• Convective heat flows into the helical coil;
• Convective heat flows into the PCM spheres;
• The conductive heat flows within the PCM spheres and their latent heat effects.

Initial runs of the model used 75 mm PCM balls. These were replaced by 40 mm balls when the impact of slow heat diffusion within the PCM was identified.

PCM were selected that provided high volumetric heat capacity and that would remain stable for at least 2000 cycles across the operating range. This resulted in the selection of hydrated salt PCM for the low temperature tank regulation at 22 °C. At higher temperatures, over 60 °C, hydrated salt stability reduces to 300 cycles (PLUSS website [44]) and organic PCM, with lower volumetric capacity but better stability, was preferred.

**Fig. 12:**
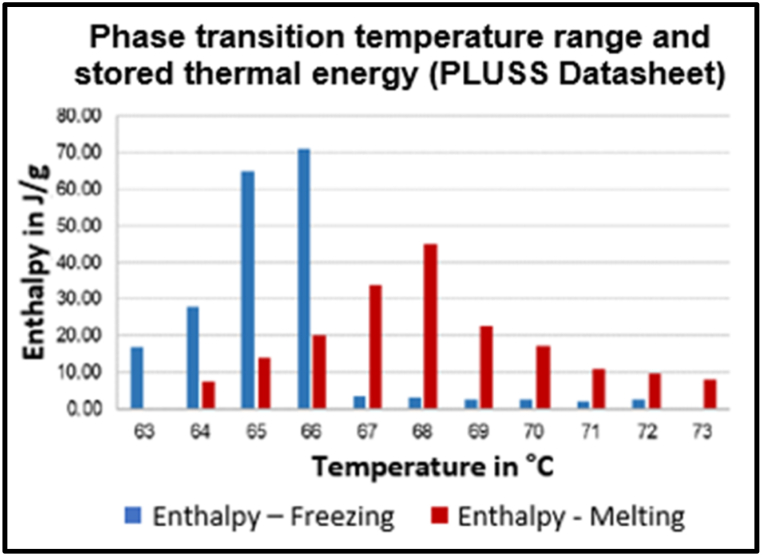
Pcm enthalpy changes (PLUSS OM65 Datasheet).

PLUSS data sheets [45] show the enthalpy released or absorbed in the PCM phase transition. An example is shown in Fig. 12.

These enthalpy changes were combined with the specific heat capacities for liquid and solid forms, to create a degree-by-degree heat capacity covering the model temperature range.

To address the heat transfer issues of PCM discussed in detail in Ding's Thermal Energy Storage [46] while staying within Simscape's simultaneous ordinary differential equation solver structure, a lumped capacitance approach was adopted. The spheres were considered in four layers, to allow for progressive melting using packed bed heat transfer correlations and assuming constant temperatures for each tank segment.

**Fig. 13:**
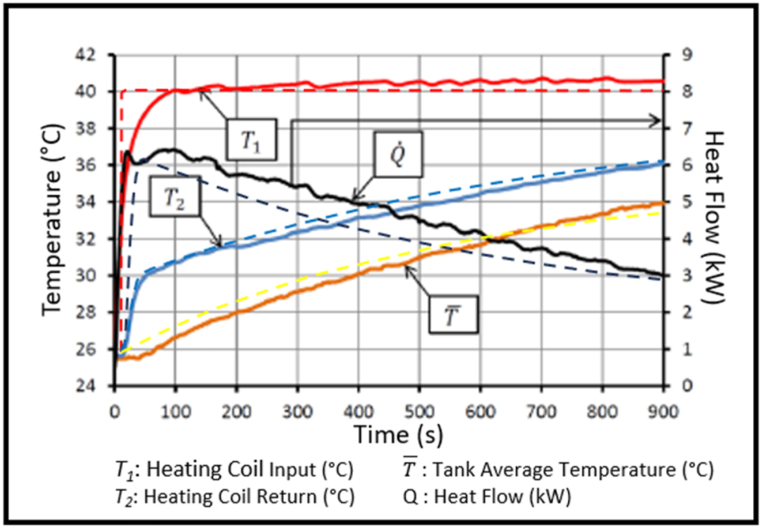
Smusz's 2016 experimental results for tube coil heat exchanger (solid) shown with the model's output (dashed).

Experimental results from Smusz [47] on a helical coil in a water storage tank were the closest data found for verification. Fins were added to the model tank coil to align with Smusz's experimental set up. The model results (dashed lines), showing how the model's tank average temperature, heating coil return temperature and heat transfer changed over time for a given heating coil input temperature, are compared with Smusz's results (solid lines) in Fig. 13.

### 2.4 Distribution and demand model

The heat distribution model was adapted from the Simscape “House Heating System” example. It was calibrated against the thermal performance of the three bedroom house defined in Lingard's 2020 retrofit study [3]. The gas fuelled boiler was replaced with the output from storage tanks heated by the heat pump.

Lingard used the EHS 2017 dwelling sample for the UK housing stock which achieved an EPC ‘D’ rating. This ‘typical’ house is semi-detached with a 91 m^2^ floor area. The outline specification of the house, provided in Lingard's paper, was supplemented with material thermal properties taken from Table 3 of the CIBSE Guide A [48].

Lingard's data shows total house energy usage 50% more than the heating system. This energy, along with solar and occupant heat, provides internal gains reducing heating system demand. Heating set temperatures were based on Shipworth et al.‘s [49] paper on UK thermostat settings. These were adjusted, allowing for internal gains, to match Lingard's annual heating system demand.

Domestic hot water consumption was taken from the Energy Saving Trust in 2008 study [13]. Hourly weather data were obtained from WorldWeatherOnline [50] for four locations spread across different parts of the UK.

## 3 Integration and optimisation

### 3.1 Heat pump

The heat pump model was tested across the ranges shown in Table 1 to identify a preferred set of operating points.

**Table 1:** Heat pump test range.

Parameter	Values
Air Source Heat Exchanger	
Ambient Temperature	−14 to 30 °C
Relative Humidity	4–95%
**Evaporator**	
Source Liquid Input Temperature	−17 to 40 °C
Source Liquid Flow^1^	0–0.85 kg/s
**Gas Cooler**	
Sink Liquid Input Temperature	14–30 °C
Sink Liquid Input Temperature	70–80 °C
**Other**	
Added Charge^2^	04. to 1.6 kg

Note 1: High value required for model initiation.

Note 2: The start-up charge was not determined.

The model ran very slowly, even after computational improvements, at only 4x faster than real time. It was necessary to replace it with a four-dimensional performance map. The source flow rate was kept constant. Over 5000 states were run, controlling.

• Compressor speed and power;
• Expansion valve orifice size;
• Target sink output temperature;
• Ambient temperature and relative humidity;
• Charge level.

Combinations were identified that.

• Maintained suction gas superheat;
• Kept all fluids within the required phase states and the compressor ratio within specification.

From these, the highest COPs were selected for each of 490 performance map input variable combinations and parallel tables produced for each heat pump output variable. A summary extract of these values is shown in Table 2.

**Table 2:** Heat pump performance table extract.

Input Variables				Output Values					
Source Input (°C)	Sink Input (°C)	Sink Output (°C)	Charge Added (kg)	Sink Flow (kg/s)	Source Output (°C)	Comp. Ratio	Heat Power (kw)	Comp. Power (kW)	COP
−17	14	70	0.4	0.011	−17.69	3.8	2.3	0.97	2.37
−5	14	70	0.4	0.013	−5.56	2.7	2.63	1.03	2.55
13	14	70	0.4	0.016	12.29	2.2	3.2	1.01	3.16
23	14	70	0.4	0.016	22.21	1.9	3.31	1	3.32
−14	26	70	1	0.016	−13.61	4.0	2.51	1.02	2.46
−5	26	70	1	0.018	−6.54	3.3	2.89	1.06	2.72
13	26	70	1	0.020	11.75	2.1	3.19	1.01	3.16
34	26	70	1	0.025	32.97	1.7	4.06	1	4.06
−14	20	80	1.6	0.012	−15.49	4.6	2.66	1.05	2.53
−5	20	80	1.6	0.013	−6.6	3.7	2.89	0.99	2.92
5	20	80	1.6	0.016	3.48	2.9	3.46	1.03	3.37
23	20	80	1.6	0.019	21.27	2.1	4.17	1.03	4.04
34	20	80	1.6	0.021	32.01	1.9	4.76	1.07	4.45

Highlighted states, which exceed compressor ratio, avoided in operation.

It can be seen from the extract that.

• COPs are within those in published results [22] and commercial CO_2_ heat pumps [10];
• Higher COPs are achieved with higher charge levels;
• Compressor ratio limits are exceeded at low source temperatures with high charge levels.

**Fig. 14:**
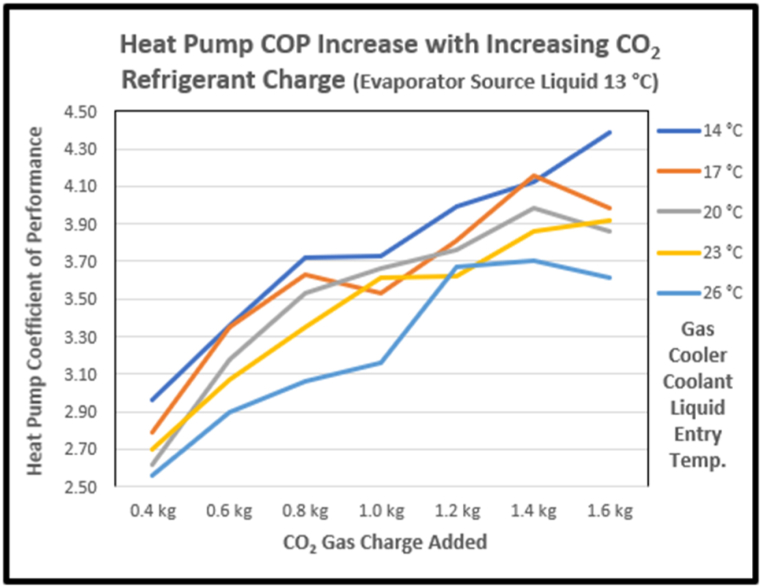
Impact of gas charge level on COP.

**Fig. 15:**
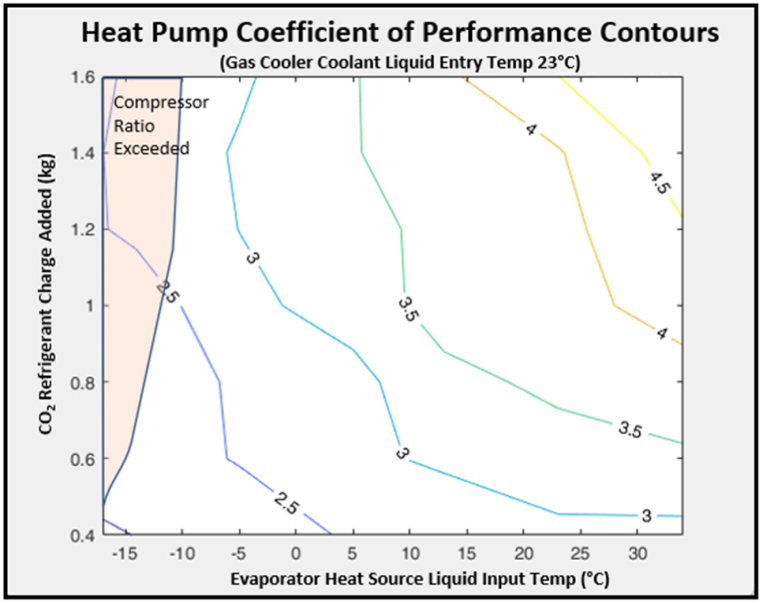
COP increasing with CO_2_ charge and evaporator heat source temperature.

Charge levels were adjusted in the model to gain performance while keeping within the compressor ratio limits. The COP increased as CO_2_ refrigerant was added up to 1.4 kg. Further increases beyond 1.4 kg saw a drop off in performance (see Fig. 14). This is in line with Aprea et al.‘s [51] experimental findings on charge optimisation. The shape of COP to added charge curves varied across different evaporator heat source temperatures. Fig. 15 shows the COP contours over the model range showing how adding charge provides 50% benefit at 25 °C source temperatures, but only about 25% at −10 °C (decreasing slightly if more than 1.4 kg of CO_2_ is added). It was further observed that model compressor pressure ratios also increased with higher charges, exceeding the modelled compressor specified maximum of 4 for lower sink input temperatures.

Rapid addition of CO_2_ through a variable orifice led to frosting, causing model failure, so transition was slowed. This will need care in the design of physical systems.

**Fig. 16:**
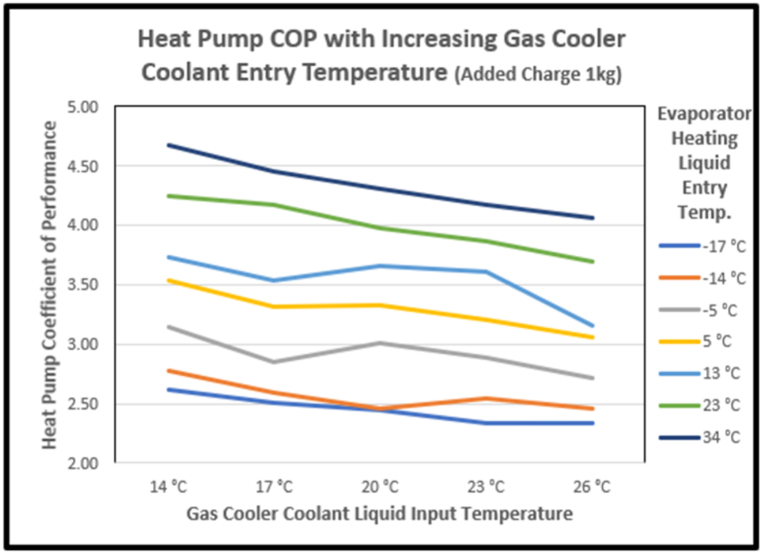
Effect on COP of gas cooler coolant liquid entry temperature.

Higher input temperatures of the coolant in the gas cooler, increased CO_2_ exit temperatures, reducing the COP in the ranges explored by about 15% (see Fig. 16). This is consistent with experimental results from Nebot-Andrés et al. [52] and the early experiments of Lorentzen discussed by Yao et al. [19].

Two further four-dimensional performance tables were created for the air source heat exchanger. One for frosting and condensation, the other for the defrost mode. Table 3 shows examples of inputs with corresponding outputs.

The conversion of the original Simscape models, which were close to the physical systems, to use the performance tables was necessary to generate full year operational data within the study period. It is recognised that this loses the transitional modelling that led Ko et al. [32] to recommend acausal software. While the transitions were slowed, using integral variables with the table output, the alignment with real world transitions has been lost. The steady state operation does continue to match the original model from which it was generated. The conversion to tables enabled the full system one year model to be run in 12 days, over 10 times faster than the original, heat pump only, model.

### 3.2 Integration and controls

The house model is based on Lingard's [3] first level of refurbishment which achieves a 66% reduction in heat loss vs the original EPC ‘D’ semi-detached solid wall house mainly through 50 mm of external wall insulation, reducing the wall ‘U’ value from 1.95 to less than 0.3 W/m^2^K.

The heat pump was limited to 1 kW input power in line with Lingard's recommendation to avoid overloading the grid (which is based on a local ADMD of 1.5 kW/household).

Three valve switched circuits on the heat pump source side connected the sub-systems.

1) Air source heat exchanger to heat pump;
2) Recycling heat from storage tanks to heat pump;
3) Recycling heat to defrost air source heat exchanger;

On the heat distribution side the valve switched circuits include.

4) Heat pump output to storage tanks;
5) DHW heated progressively through coils in the heat pump storage tanks into the DHW storage tank and thence to distribution in accordance with DHW demand pattern;
6) DHW storage tank temperature boosted through coil in heat pump hot tank if its temperature falls beneath the legionnaire protection 55 °C;
7) Pulsed distribution, alternately from hot storage tank and middle tank as respective temperatures permit, to building radiators;
8) Recycling heat from middle tank and cool tank to support defrosting and heat recycling to maintain heat pump return temperatures close to 22 °C.

**Fig. 17:**
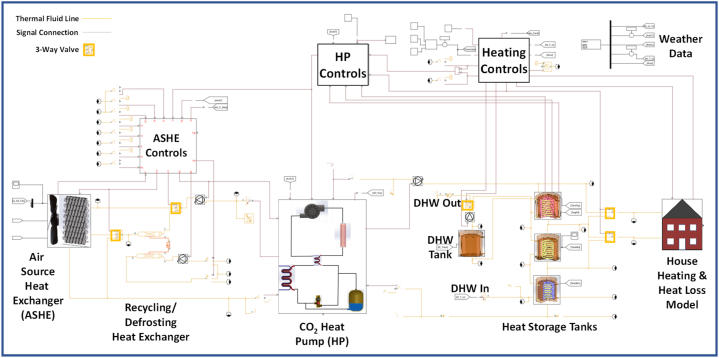
Overall simscape model.

Fig. 17 shows the complete model. The heat pump and ASHE use the performance tables with linear interpolation and integral smoothing between points.

### 3.3 System performance

The study objective was to achieve a COP of over 3 on a full year run. To achieve a full year run within a week, and so have opportunity to adjust variables and explore differences, the model had to run around 50 times faster than real time. Several computational methods were used to achieve this.

• Replacing complex sub-systems with performance tables (heat pump and ASHE);
• Reducing stratification layers from 5 to 3 in the heat storage tanks;
• Selecting less computational intensive correlations, eg Oro [53] convection around packed bed spheres replaced with Ahmed and Yovanovich (from Will [54]);
• Smoothing transitions, especially around valve change of states;
• Eliminating oscillations by separating on/off threshold variables.

These changes, combined with running month simulations in parallel, completed a full year run within 2 days. The most significant cost of these changes was the loss of transitions resulting from the move to performance tables.

## 4 Results and discussion

### 4.1 Coefficient of performance

**Fig. 18:**
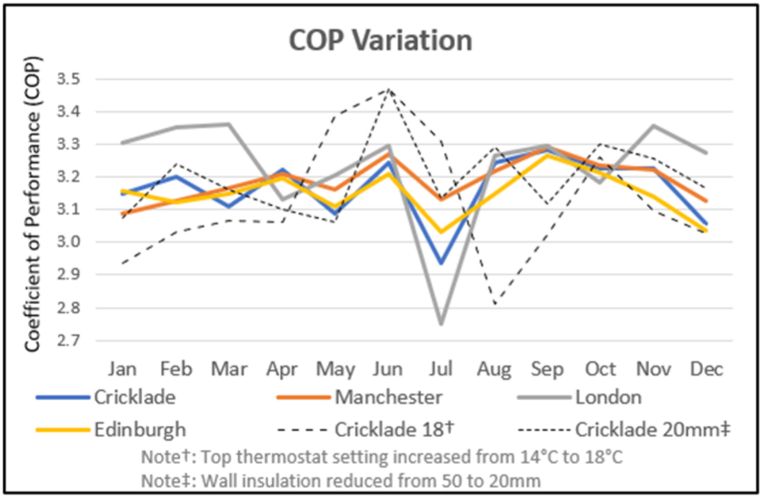
COP for different UK Locations, Thermostat Settings and Insulation Levels.

The model was run for full years using weather data from four UK locations, to cover different climates. The building used Lingard's data [3] adopting 50 mm of solid wall insulation. This level of insulation halved the heat demand in Lingard's model from the original EPC ‘D’ based model to 47.4 kWh/m^2^/yr in the 91 m^2^ building. To match these heating demand figures with the same insulation, required maximum thermostat settings of 14 °C. While it is recognised that solar and internal energy gains might add 3–4 °C, this thermostat maximum setting was considered low. The model was therefore also run with the settings increased to 18 °C. The system COPs are shown in Fig. 18. This also includes a run with the solid wall insulation reduced from 50 mm to 20 mm for comparative purposes.

The activity times for the heat pump, recycling and defrosting varied across seasons. The defrosting and low DHW inlet temperatures helped maintain the gas cooler input temperatures in the winter. Even without heating, some recycling was required in the summer. Efficiency improvements expected from higher ASHE temperatures in the summer were negated by high DHW inlet temperatures, and higher, in proportion to total heat provided, heat loss from storage.

System performance was found to be sensitive to the target temperature of the low temperature storage tank feeding the gas cooler. A summer recycling threshold of 22 °C was used, almost eliminating the need for heat recycling except in southern UK in July. The cooler winter water enabled a threshold of 18 °C to be set with the water assisting gas cooler input temperature control.

The July dip in system COP, most pronounced in London, correlated with higher cold water input temperatures (>20 °C) and low water usage (5% less than August). Heat pump COP increased from 3.5 in the winter to 3.7 in the summer. However, the reduction in gas cooler subcooling from the cold water input, the absence of space heating demand, led to increased recycling mode operation to achieve the targeted gas cooler exit temperatures.

Conversely, in winter, London COPs exceeded other locations as low cold water temperatures and London's higher ambient temperatures assisted the DHW to space heating ratio. This is in line with Brodal and Jackson's [27] findings.

**Fig. 19:**
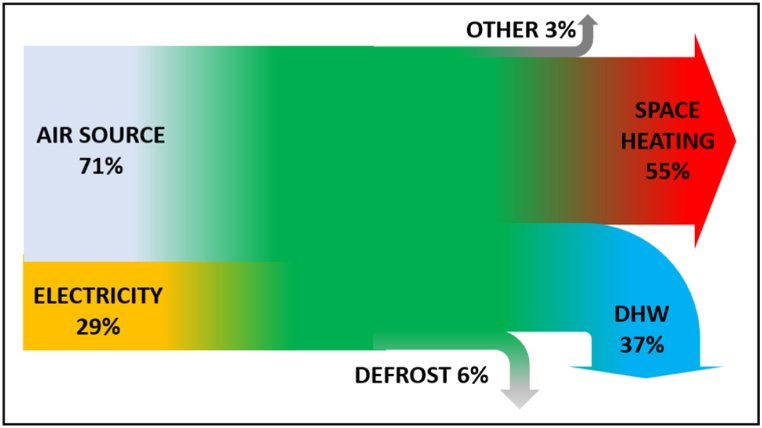
System energy inputs and out-flows.

The results show the annual total heating and DHW requirement being met with COP of between 3.14 and 3.27. This was maintained even as insulation level were reduced to 20 mm and internal temperatures increased 4 °C, comparing favourably with a 2.41 UK average for air source heat pumps (SPFH4 from Lowe et al. [55]). Fig. 19 shows the system energy flows.

#### 4.1.1 Pulsing

No benefit was found from radiator pulsing. This may be because the defrosting was using most of the medium and low temperature heat.

#### 4.1.2 Domestic hot water inlet temperature

**Fig. 20:**
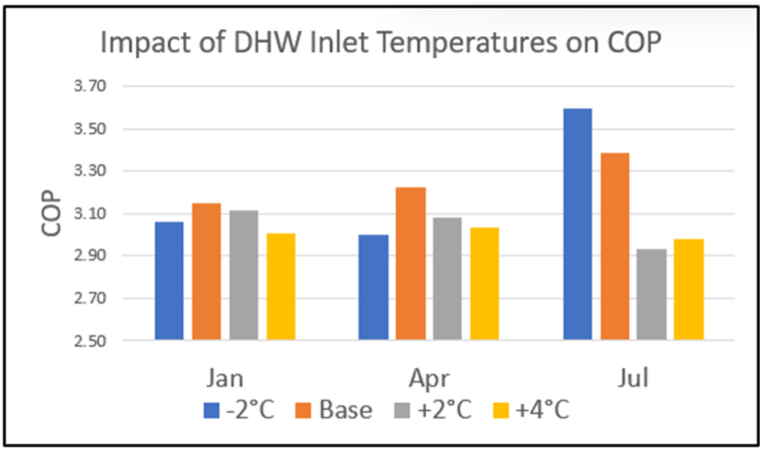
Impact of DHW on COP.

While DHW requires energy to raise its temperature, cold inlet water helps keep the gas cooler inlet temperature down, raising efficiency. Fig. 20 shows the impact of changes in DHW temperatures on COP. An adverse effect is seen in July if water temperatures exceed 21 °C. This confirms the need to control gas cooler inlet temperatures.

#### 4.1.3 Phase change materials

The PCM stored energy peaked at 52% of the total in the top tank, while occupying 46% of the volume. Removing the PCM from the system increased the stored energy as heat transfer liquid, that was already heated, occupied the full volume. Low PCM thermal conductivity slows energy absorption which, even adopting 40 mm balls with higher surface to volume ratios than the standard 75 mm balls, is too slow to realise PCM's benefits in this system. PCM would need to achieve faster internal energy transfer than that exhibited by the homogenous PCM materials modelled is this study to add significant benefit.

While the latent heat of the PCM transition could potentially store 4 times as much as the equivalent volume of water/glycol, only a small volume of PCM at the surface of the sphere melted or solidified within the timescales of the tank temperature changes in this system.

#### 4.1.4 Heat pump modes of operation

**Fig. 21:**
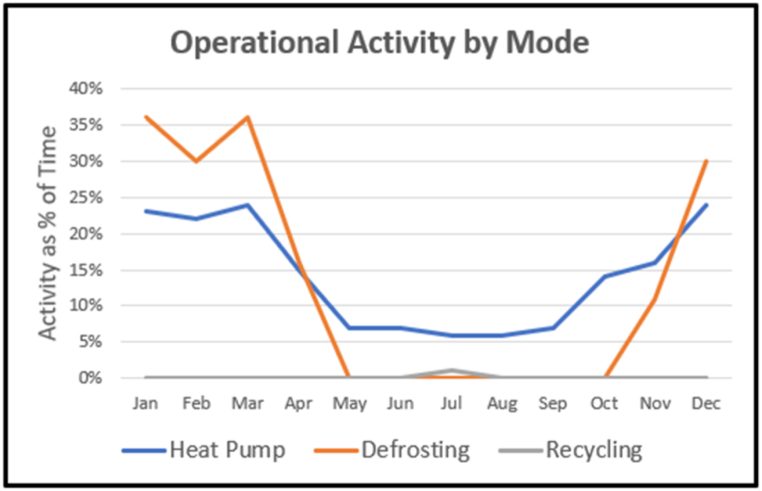
Heat pump mode activity levels.

Fig. 21 shows the proportion of time that the heat pump was in each of its operational modes. Winter defrosting was in operation for up to a third of time. The heat pump was never used for more than 25% of time. Recycling was only significant in the summer, reaching 16% of the heat pump operation time.

#### 4.1.5 Defrost

No comparative defrost energy usage data was found for alternative defrost methods. Studies focused on complexity, cost and speed of defrost. Combining defrosting with the recycling, to perform heat pump input temperature regulation as well as defrosting, adds little cost or complexity. Operating the defrost when the heat pump is off, even with low frost thicknesses, avoids the interruption or the indoor cooling that the widely implemented reverse cycle defrost causes. However, it was found that using the middle and low temperature storage tanks only, was not sufficient to eliminate thick frost. Heat from the hot tank may also be required (this was not tested within this study). Defrost efficiencies are lower with thin frost layers, in line with da Silva et al.‘s [36] efficiency equation applied in the model's operating range (m’’ < 0.06 kg/m^2^):

η=(1.285–0.5m’’)m’’

where m’’ = kg of frost/m^2^ of surface.

For the frost thicknesses below 1 mm identified in this study, this gives very low efficiencies and, at a maximum of 5% efficiency, is outside the experimental range explored by da Silva et al. which was focused on 10% and above.

**Fig. 22:**
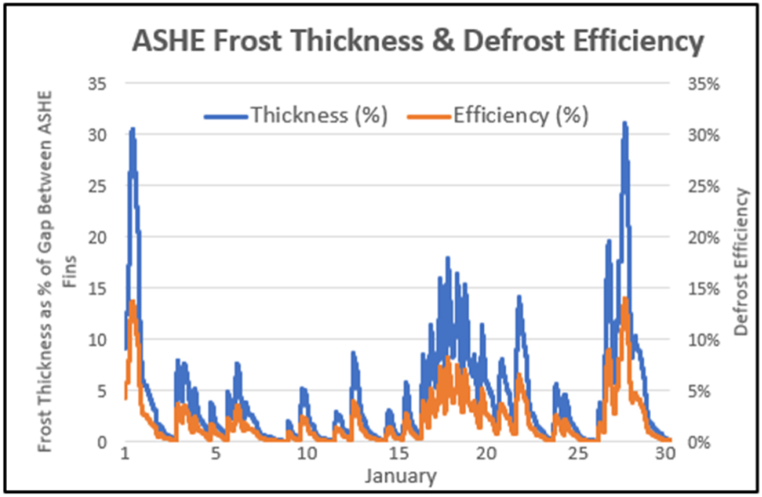
January frost thickness & defrost efficiency.

Fig. 22 shows the frost thicknesses for January as % of the gap between heat exchanger fins. Defrosting of thin layers is less efficient, and, at these efficiencies, uses more low temperature heat than is required to cool the gas cooler inlet.

The low temperature heat of the middle and lower tanks must be supplemented by heat from the hot tank if frost layers are to be permitted to thicken and defrosting is limited to non-heat pump operation periods. The defrost efficiency formula needs to be calibrated for the thin frost layers and low temperature heat supply involved in this system.

Defrosting was active for 13% of time (36% in January) consuming 6% of the annual heat pump output power.

#### 4.1.6 Recycling

Recycling, active when gas cooler inlet temperatures are too high, uses low exergy fluid from the lower temperature tanks as a source for the heat pump. It is necessary when all other means of cooling the gas cooler inlet have been exploited. While defrosting and DHW inlet water performed this function in the winter, up to 6% of heat pump output energy had to be recycled in July when water inlet temperatures were highest. This lowered the July COPs.

#### 4.1.7 Solar

**Fig. 23:**
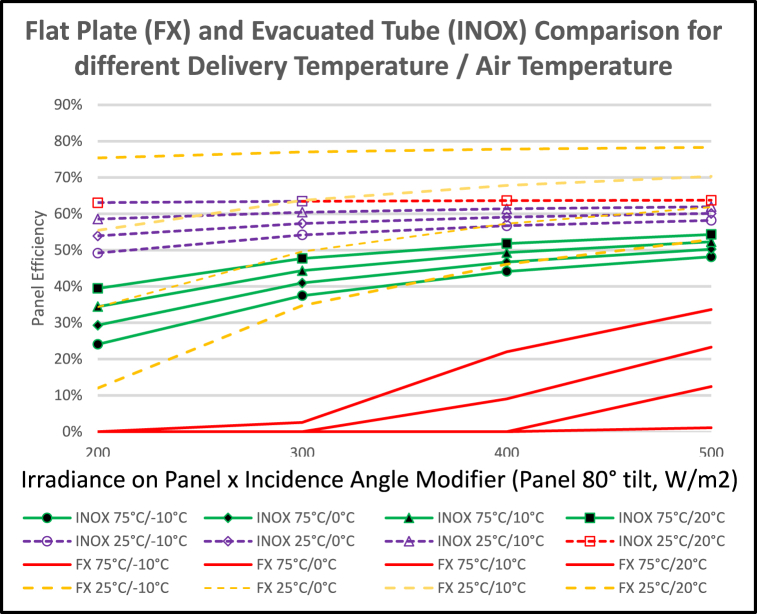
Comparison of flat plate and evacuated tube solar collector efficiencies.

The impact of solar panels was explored. It was found that, when DHW input temperatures are over 21 °C, recycling activity increases to over 15% of heat pump operation time. Solar photovoltaic panels, with an efficiency of 15–20% [55], powering the heat pump with a July COP of 3, can achieve a 45–60% benefit. Thermal collectors, directly heating the hot tank and so avoiding the need for recycling, can achieve efficiencies up to 58%. They can integrate easily, with lower costs than a dedicated PV system with inverter. Fig. 23 considers the relative merits of flat plate and evacuated tube collectors, connecting to high or low temperature tanks.

Initial investigations suggested that a 60% improvement in COP could be achieved with evacuated tubes directly heating the high temperature storage tank. Optimising collector tilt angles could increase performance, but the aesthetic impact of non-flush wall or roof mounts must be considered.

#### 4.1.8 System improvement

While model subsystems were calibrated, where possible, with published studies, and the heat pump COP is comparable with manufacturer data, it is recognised that the model has uncalibrated heat exchangers whose sizing was determined without direct reference to known equipment. These were constructed using Simscape pipe components using single surface temperatures rather than the varying ones seen in real heat exchangers. These include.

• Heat pump gas cooler, evaporator and internal heat exchanger;
• Air source and recycling heat exchangers.

While multiple stages addressed some of this problem, the model accuracy has not been determined. The energy consumed by the air source fan and the circulation pumps has been considered, the heat pump compressor energy usage incudes its isentropic efficiency, however, the electrical or mechanical efficiencies were not modelled. A 95% combined efficiency adjustment has been applied to cover this.

Improvement opportunities exist that have not yet been included in the model.

• Heat exchanger optimisation;
• Replacing the expansion valve with an ejector;
• Higher efficiency compressor;
• Frost retarding coatings and geometries;
• Alternative control strategies, eg lower top temperatures in warmer months when heating is less demanding;

The model used a separate heat exchanger for the defrost and recycling heat transfer with a heat transfer fluid carrying the heat between this heat exchanger, the air source heat exchanger and the heat pump evaporator. An alternative implementation is to directly heat the heat pump evaporator in the air source heat exchanger, and integrate the defrost and recycling heat transfer fluid flow into a secondary pipe system built into the air source heat exchanger fins. While some recycling heat would be lost to the outside air (the fan would be off and there would be limited air circulation across the heat exchanger during recycling and defrosting) it would reduce the number of heat transfer stages required, each of which involves a drop in temperature, affecting heat pump efficiency.

### 4.2 Economic & emissions impact

While the UK Government's Heat and Buildings Strategy failed to produce immediate changes, some incentives are now emerging. The 2023 increase of heat pump grants to £7500 per installation and VAT reductions on insulation materials offer some assistance. Meanwhile, the recent sudden increases in electricity and wholesale gas prices have changed the economic environment.

In September 2022 the UK Government set the energy price cap at £2500, implying 34p/kWh for electricity and 10.3p for gas [56]. Prices appear to have stabilized close to these prices after a period of extreme volatility. At these rates, the system COP of 3.15 only just achieves operational cost breakeven.

**Fig. 24:**
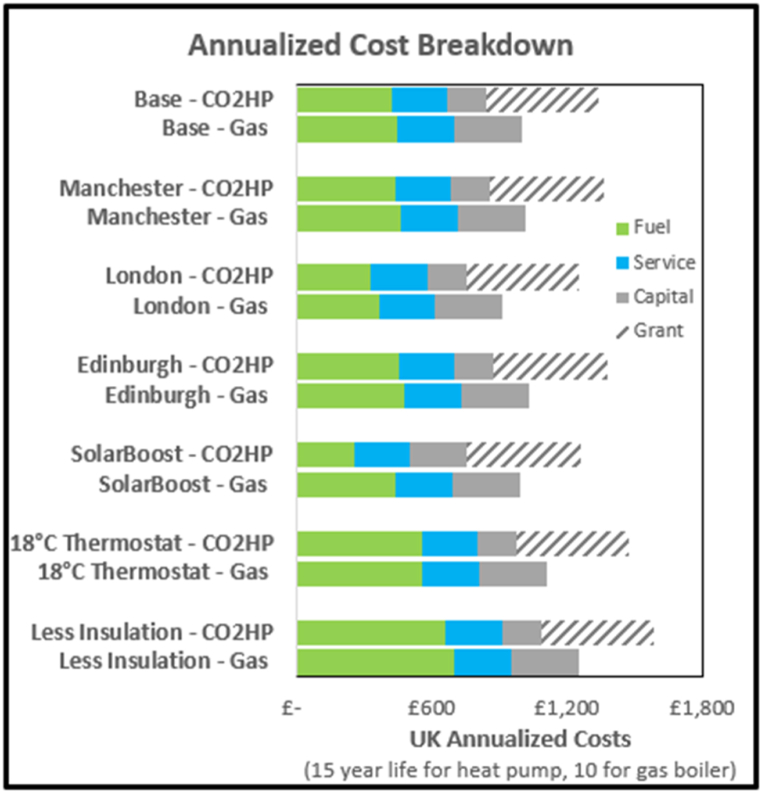
Comparison of cost structure across scenarios.

The capital costs of the system considered, without the PCM, are estimated at £1600 more than current heat pumps. The capital cost penalty over a gas boiler, calculated at £7100 for the modelled system, is fully covered by the recently increased UK Government grant. Assuming 15 year asset lives in line with NESTA [31], results in a levelized cost of energy delivered (LCOE) of 21–24p/kWh for the modelled system heat supplied (gas: 26p/kWh) with capital costs most significant. See Fig. 24 for comparisons which also shows the impact of the grant support.

**Fig. 25:**
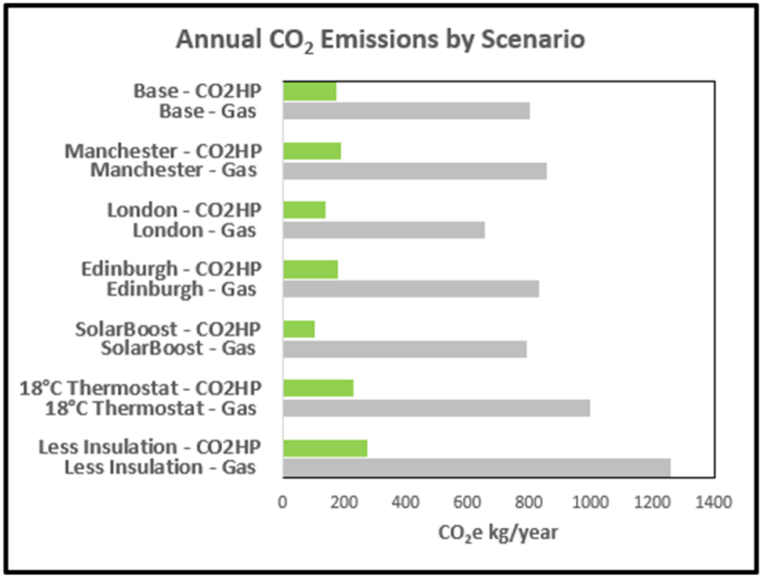
Comparison of annual CO2 emissions by scenario.

The carbon intensity of the UK electricity grid is expected to drop from 213 to about 50 gCO_2_e/kWh from 2022 to 2032 [57], while the carbon intensity of gas will stay constant at 185 gCO_2_e/kWh. Table 3 shows the carbon intensities applied per kWh for the electricity consumed vs a notional 90% efficient condensing gas boiler. On this basis, 9.45 tonnes of CO_2_ emissions are saved over fifteen years in the Cricklade base case. Operational carbon neutrality will be approached with the full decarbonization of the electricity supply. Operational emissions will then be confined to leakage (15% lifetime leakage assumption on an estimated charge of 4 kg = 0.6 kgCO_2_e over 15 years). Embedded emissions elimination is dependent on carbon neutral steel, manufacturing and transport. Current figures for these embedded emissions have not been included in this analysis. Fig. 25 shows the comparison across modelled variants.

**Table 3:**
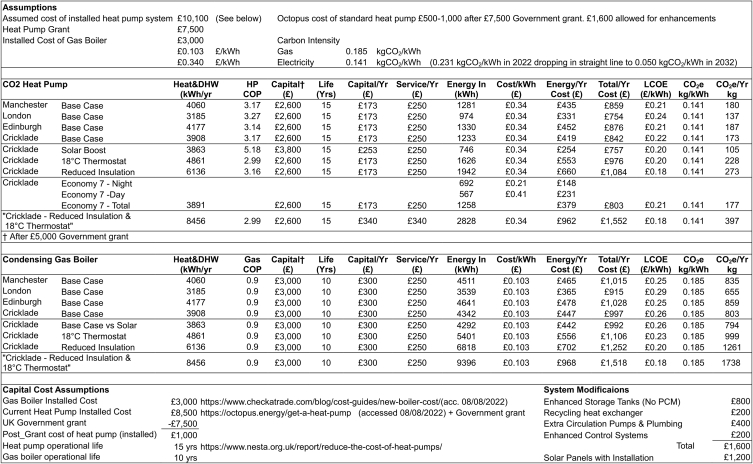
Economic Analysis.

Operating the heat pump at night, when ambient temperatures are lower, but when Economy7 tariffs can be utilized, supplied morning heat at lower cost, however, the higher daytime rates negated the overnight benefit. The LCOE of 21p/kWh, similar to using the standard tariff.

The marginal investment of £1200 in solar collectors justifies itself, achieving simple payback in 6 years, with a system LCOE of 20p/kWh.

The low level of usage in a single well insulated dwelling results in long investment payback periods. In houses where only medium or low levels of insulation are possible (eg. due to conservation orders) the heat pump becomes more economically advantageous. A 20 mm wall insulation house, with solar collectors and 2 °C of internal gains, achieved a LCOE of 18p/kWh.

Against the base case, the CO_2_ heat pump can just match a gas boiler LCOE, the increased technological and economic risks and dependence on the government grant imply that the case is not yet compelling. A summary of the economic analysis is shown in Table 3.

### 4.3 Observations

The model predicts system annual COPs of 3.14–3.27, well above the reported 2.41 [48] for 2017 heat pumps and surpassing the UK Climate Change Committee heat pump COP forecast of 3.0 by 2030 [58].

Control systems are critical to achieving the system benefits modelled, particular sensitivities to bottom tank temperatures and defrost controls were observed.

Defrost efficiencies at low heating temperatures on thin layers need to be explored. The model is operating outside the range of identified experimental research. Use of the hot tank heat at the end of the defrost cycle may be required to dry the ASHE prior to restarting the heat pump.

The optimisation of the heat pump is situation dependent. Increased building insulation creates opportunity for lower radiator temperatures, but adds cost and installation inconvenience.

Exploring this relationship could inform decisions on different levels of insulation made possible by the temperature range of the CO_2_ heat pump. Recent research [59] into using ejectors for expansion suggests performance improvements are possible, although the impact of capital cost increases would need to be assessed.

The recycling mode cools the lower tank while heating the tanks above. Switching radiator circulation to use the lower tank provides and option for summer cooling. The heat extracted being used for water heating and/or dumped externally.

Trade-offs, to achieve practical model run times, have resulted in the model departing from pure representations of physical devices. It is recognised that significant model changes were made following heat pump model validation (expanding capacity and altering charge levels). These weaken the value of the validation. The results should therefore be considered as indicative.

The advantages of the Simscape acausal software approach recommended by Ko et al. [32] were only found in the sub-system models. In these, the proximity to physical systems provided insight to the underlying processes and their interrelationships. Processing time and run time failures increased rapidly with model size, as sub-systems were integrated, reducing the benefit of this software.

Multiple scenarios and parameter settings were run, with optimisation incremental and subjective. Artificial neural network techniques, as used by Dai et al. [26] could improve the parameter optimisation and overall performance.

## 5 Conclusion

### 5.1 COP improvement

Transcritical CO_2_ heat pump systems can achieve seasonal COPs in excess of 3.1 in an EPC ‘D’ UK house with 50 mm external wall insulation added. The system approach has overcome the gas cooler temperature issue preventing the use of CO_2_ heat pumps for domestic space heating. The novel defrosting system, utilising radiator return heat, improved heat pump efficiency. However, this provided no assistance in summer when high DHW inlet temperatures adversely affected performance.

### 5.2 Solar boost

The addition of south facing, near vertically mounted, solar thermal collectors can boost efficiency, replacing the use of the heat pump in the summer when DHW inlet temperatures are high, increasing the system annual COP to 5.1.

With the solar collectors, and current UK Government grants and energy prices, the CO2 heat pump system can achieve a levelized cost of energy (LCOE) of 20p/kWh, 23% lower than the 26p/kWh for a replacement condensing gas boiler. Capital costs are most significant, with the grant critical to cost savings.

### 5.3 CO_2_ emission savings

The modelled emissions savings for the semi-detached dwelling of the transition from a gas boiler to a transcritical CO_2_ heat pumps are 6.3 tonneCO_2_e over 10 years, increasing to 7 tonneCO_2_e with the solar collectors.

### 5.4 Low insultation situations

Investigations with reduced insulation, showed that the 1 kW input power heat pump could maintain room temperatures in houses where high levels of retrofit insulation were not possible. Improved LCOE arising from the higher usage (18p/kWh) suggest that the transcritical CO_2_ heat pumps may be particularly effective in this context.

## Data availability

The principal results data can be found at https://www.dropbox.com/scl/fo/9lssdybg6tdik8y4knxd0/h?rlkey=j35o9cym4z6vfkalhu3rmrp6z&dl=0. The models, simulation data inputs and outputs can also be made available.

Multiple model runs were used to generate the data at both sub-system and system level. Explanations can be provided to ensure the most appropriate data is provided. To obtain further data please contact the first mentioned author.

## CRediT authorship contribution statement

**W. Lambert:** Writing – original draft, Writing – review & editing, Methodology, Investigation. **Z. Dehouche:** Supervision.

## Declaration of competing interest

The authors declare that they have no known competing financial interests or personal relationships that could have appeared to influence the work reported in this paper.
